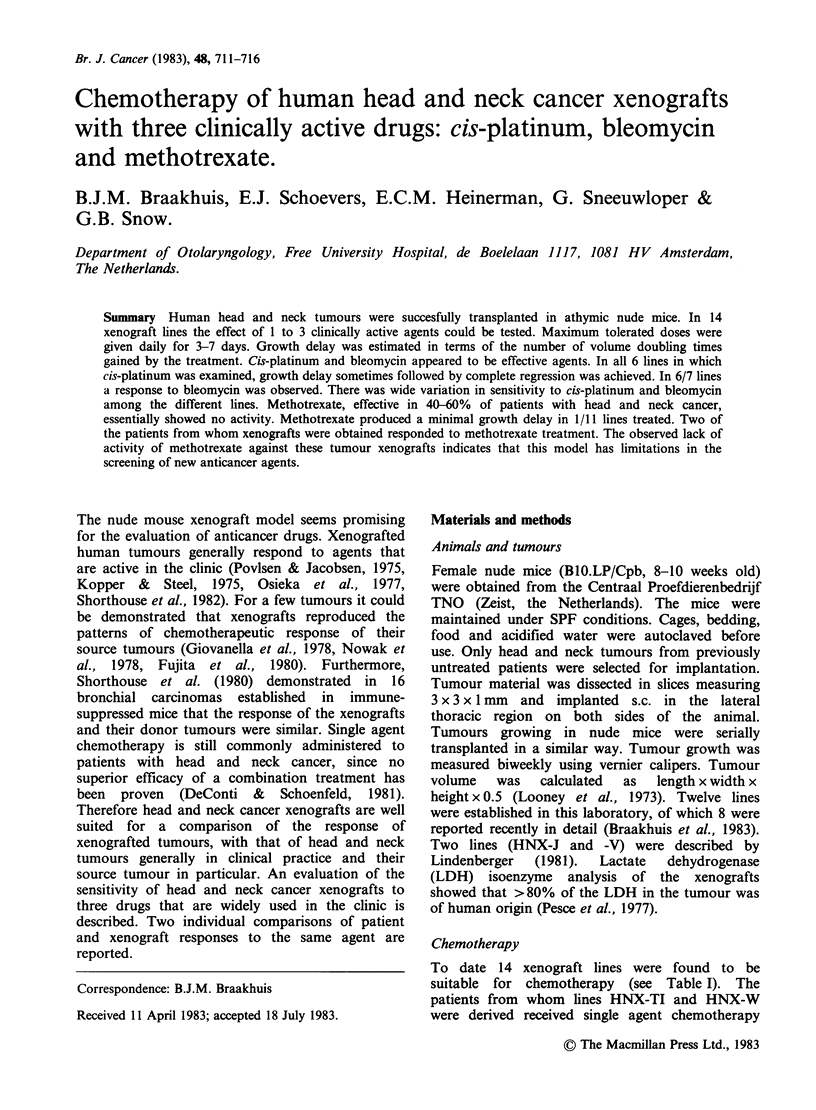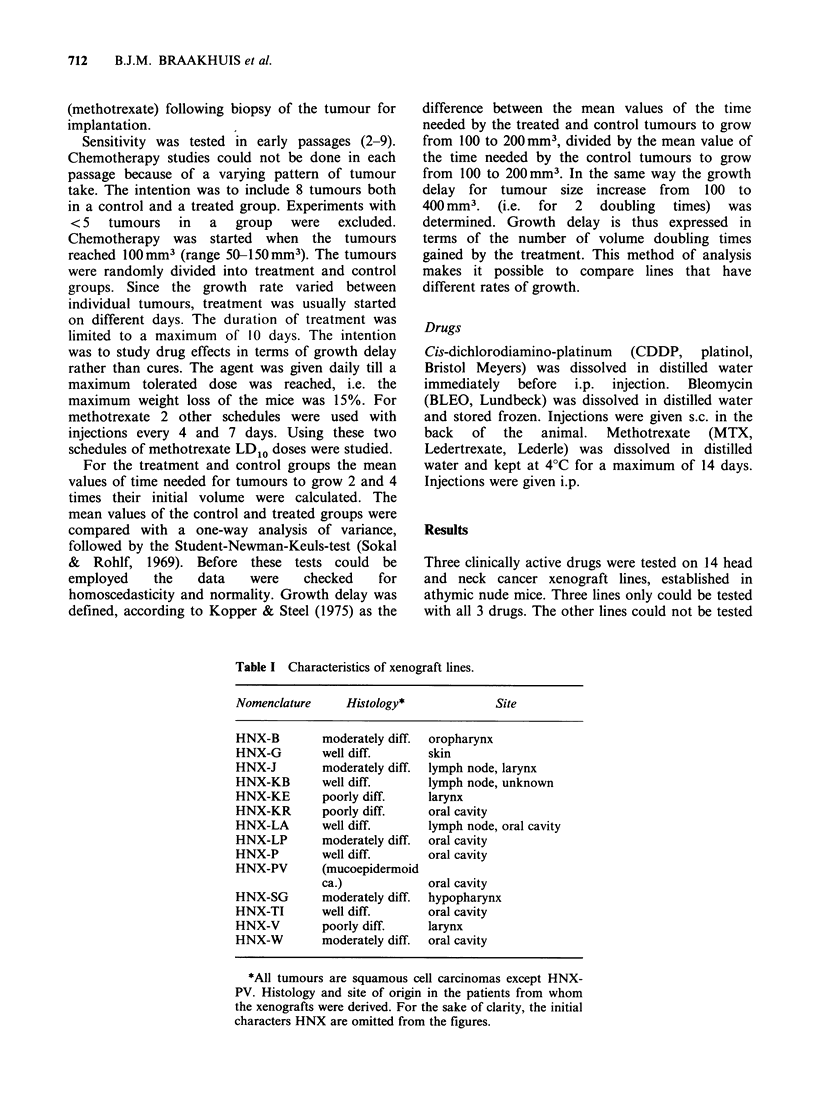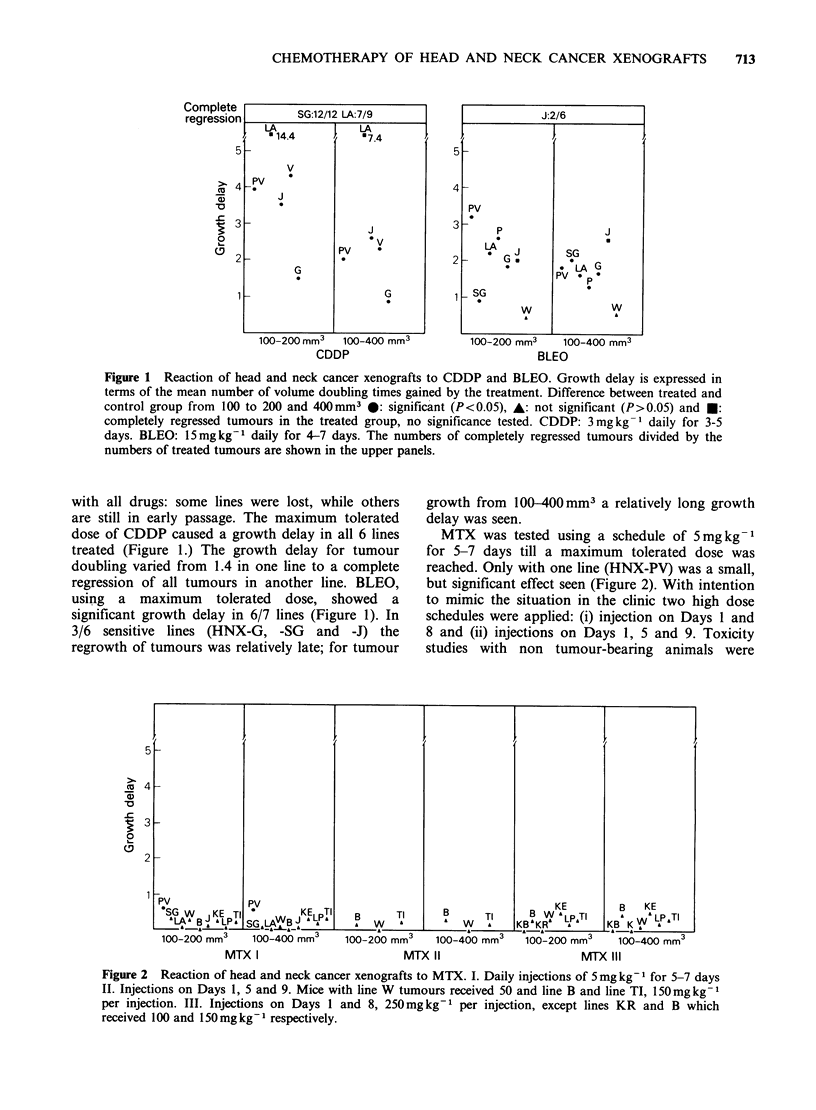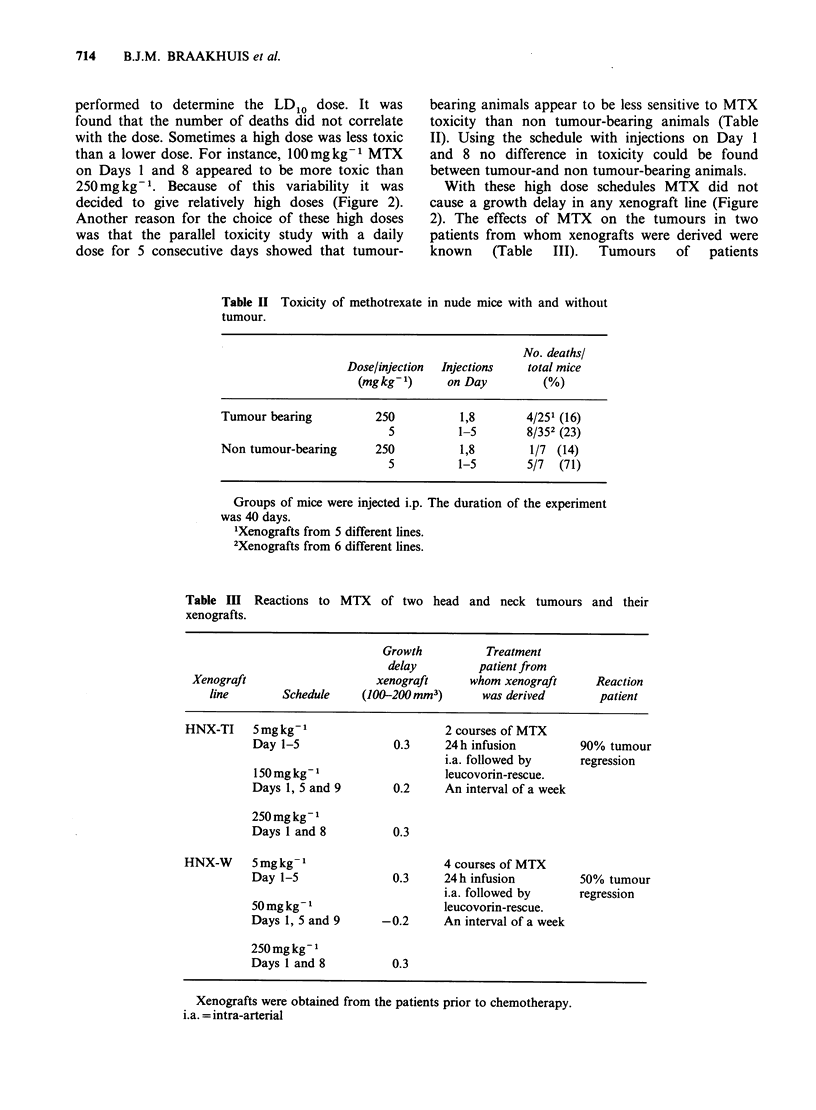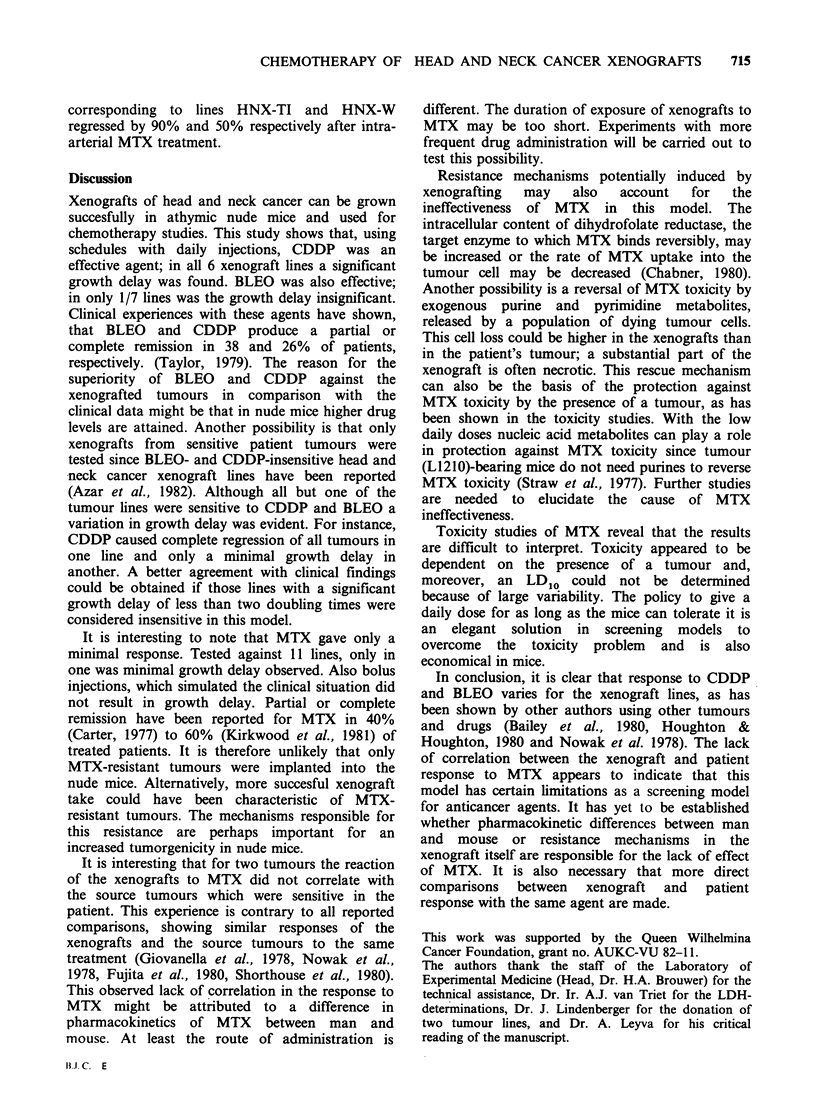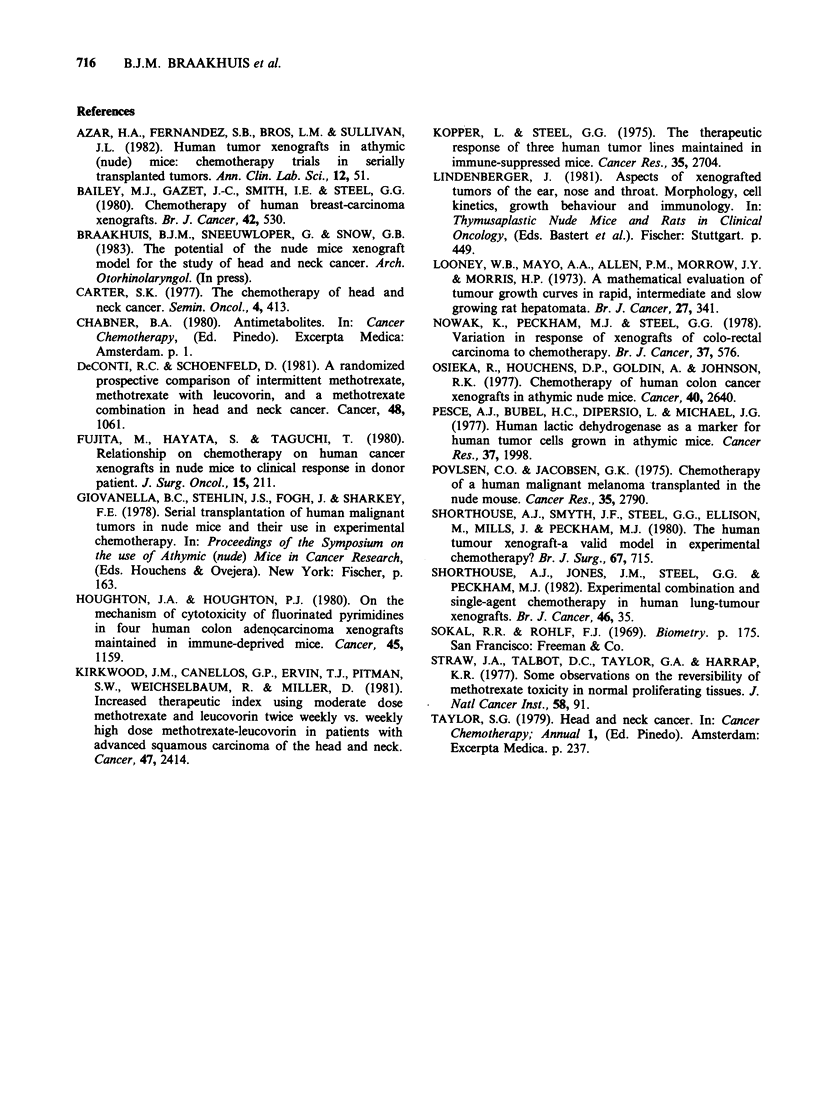# Chemotherapy of human head and neck cancer xenografts with three clinically active drugs: cis-platinum, bleomycin and methotrexate.

**DOI:** 10.1038/bjc.1983.254

**Published:** 1983-11

**Authors:** B. J. Braakhuis, E. J. Schoevers, E. C. Heinerman, G. Sneeuwloper, G. B. Snow

## Abstract

Human head and neck tumours were successfully transplanted in athymic nude mice. In 14 xenograft lines the effect of 1 to 3 clinically active agents could be tested. Maximum tolerated doses were given daily for 3-7 days. Growth delay was estimated in terms of the number of volume doubling times gained by the treatment. Cis-platinum and bleomycin appeared to be effective agents. In all 6 lines in which cis-platinum was examined, growth delay sometimes followed by complete regression was achieved. In 6/7 lines a response to bleomycin was observed. There was wide variation in sensitivity to cis-platinum and bleomycin among the different lines. Methotrexate, effective in 40-60% of patients with head and neck cancer, essentially showed no activity. Methotrexate produced a minimal growth delay in 1/11 lines treated. Two of the patients from whom xenografts were obtained responded to methotrexate treatment. The observed lack of activity of methotrexate against these tumour xenografts indicates that this model has limitations in the screening of new anticancer agents.


*Br. J. Cancer* (1983), **48**, 711–716

### Chemotherapy of human head and neck cancer xenografts with three clinically active drugs: *cis*-platinum, bleomycin and methotrexate.

B.J.M. Braakhuis, E.J. Schoevers, E.C.M. Heinerman, G. Sneeuwloper & G.B. Snow.

*Department of Otolaryngology, Free University Hospital, de Boelelaan 1117, 1081 HV Amsterdam, The Netherlands.*

**Summary** Human head and neck tumours were succesfully transplanted in athymic nude mice. In 14 xenograft lines the effect of 1 to 3 clinically active agents could be tested. Maximum tolerated doses were given daily for 3–7 days. Growth delay was estimated in terms of the number of volume doubling times gained by the treatment. *Cis*-platinum and bleomycin appeared to be effective agents. In all 6 lines in which *cis*-platinum was examined, growth delay sometimes followed by complete regression was achieved. In 6/7 lines a response to bleomycin was observed. There was wide variation in sensitivity to *cis*-platinum and bleomycin among the different lines. Methotrexate, effective in 40–60% of patients with head and neck cancer, essentially showed no activity. Methotrexate produced a minimal growth delay in 1/11 lines treated. Two of the patients from whom xenografts were obtained responded to methotrexate treatment. The observed lack of activity of methotrexate against these tumour xenografts indicates that this model has limitations in the screening of new anticancer agents.

The nude mouse xenograft model seems promising for the evaluation of anticancer drugs. Xenografted human tumours generally respond to agents that are active in the clinic (Povlsen & Jacobsen, 1975, Kopper & Steel, 1975, Osieka *et al.*, 1977, Shorthouse *et al.*, 1982). For a few tumours it could be demonstrated that xenografts reproduced the patterns of chemotherapeutic response of their source tumours (Giovanella *et al.*, 1978, Nowak *et al.*, 1978, Fujita *et al.*, 1980). Furthermore, Shorthouse *et al.* (1980) demonstrated in 16 bronchial carcinomas established in immune-suppressed mice that the response of the xenografts and their donor tumours were similar. Single agent chemotherapy is still commonly administered to patients with head and neck cancer, since no superior efficacy of a combination treatment has been proven (DeConti & Schoenfeld, 1981). Therefore head and neck cancer xenografts are well suited for a comparison of the response of xenografted tumours, with that of head and neck tumours generally in clinical practice and their source tumour in particular. An evaluation of the sensitivity of head and neck cancer xenografts to three drugs that are widely used in the clinic is described. Two individual comparisons of patient and xenograft responses to the same agent are reported.

Correspondence: B.J.M. Braakhuis

Received 11 April 1983; accepted 18 July 1983.

### Materials and methods

### *Animals and tumours*

Female nude mice (B10.LP/Cpb, 8–10 weeks old) were obtained from the Centraal Proefdierenbedrijf TNO (Zeist, the Netherlands). The mice were maintained under SPF conditions. Cages, bedding, food and acidified water were autoclaved before use. Only head and neck tumours from previously untreated patients were selected for implantation. Tumour material was dissected in slices measuring $3 \times 3 \times 1$ mm and implanted s.c. in the lateral thoracic region on both sides of the animal. Tumours growing in nude mice were serially transplanted in a similar way. Tumour growth was measured biweekly using vernier calipers. Tumour volume was calculated as length $\times$ width $\times$ height $\times$ 0.5 (Looney *et al.*, 1973). Twelve lines were established in this laboratory, of which 8 were reported recently in detail (Braakhuis *et al.*, 1983). Two lines (HNX-J and -V) were described by Lindenberger (1981). Lactate dehydrogenase (LDH) isoenzyme analysis of the xenografts showed that $>80\%$ of the LDH in the tumour was of human origin (Pesce *et al.*, 1977).

### *Chemotherapy*

To date 14 xenograft lines were found to be suitable for chemotherapy (see Table I). The patients from whom lines HNX-TI and HNX-W were derived received single agent chemotherapy

© The Macmillan Press Ltd., 1983

(methotrexate) following biopsy of the tumour for implantation.

Sensitivity was tested in early passages (2–9). Chemotherapy studies could not be done in each passage because of a varying pattern of tumour take. The intention was to include 8 tumours both in a control and a treated group. Experiments with <5 tumours in a group were excluded. Chemotherapy was started when the tumours reached 100 mm³ (range 50–150 mm³). The tumours were randomly divided into treatment and control groups. Since the growth rate varied between individual tumours, treatment was usually started on different days. The duration of treatment was limited to a maximum of 10 days. The intention was to study drug effects in terms of growth delay rather than cures. The agent was given daily till a maximum tolerated dose was reached, i.e. the maximum weight loss of the mice was 15%. For methotrexate 2 other schedules were used with injections every 4 and 7 days. Using these two schedules of methotrexate $LD_{10}$ doses were studied.

For the treatment and control groups the mean values of time needed for tumours to grow 2 and 4 times their initial volume were calculated. The mean values of the control and treated groups were compared with a one-way analysis of variance, followed by the Student-Newman-Keuls-test (Sokal & Rohlf, 1969). Before these tests could be employed the data were checked for homoscedasticity and normality. Growth delay was defined, according to Kopper & Steel (1975) as the difference between the mean values of the time needed by the treated and control tumours to grow from 100 to 200 mm³, divided by the mean value of the time needed by the control tumours to grow from 100 to 200 mm³. In the same way the growth delay for tumour size increase from 100 to 400 mm³. (i.e. for 2 doubling times) was determined. Growth delay is thus expressed in terms of the number of volume doubling times gained by the treatment. This method of analysis makes it possible to compare lines that have different rates of growth.

### *Drugs*

*Cis*-dichlorodiamino-platinum (CDDP, platinol, Bristol Meyers) was dissolved in distilled water immediately before i.p. injection. Bleomycin (BLEO, Lundbeck) was dissolved in distilled water and stored frozen. Injections were given s.c. in the back of the animal. Methotrexate (MTX, Ledertrexate, Lederle) was dissolved in distilled water and kept at 4°C for a maximum of 14 days. Injections were given i.p.

### Results

Three clinically active drugs were tested on 14 head and neck cancer xenograft lines, established in athymic nude mice. Three lines only could be tested with all 3 drugs. The other lines could not be tested

**Table I** Characteristics of xenograft lines.

| *Nomenclature* | *Histology** | *Site* |
|---|---|---|
| HNX-B | moderately diff. | oropharynx |
| HNX-G | well diff. | skin |
| HNX-J | moderately diff. | lymph node, larynx |
| HNX-KB | well diff. | lymph node, unknown |
| HNX-KE | poorly diff. | larynx |
| HNX-KR | poorly diff. | oral cavity |
| HNX-LA | well diff. | lymph node, oral cavity |
| HNX-LP | moderately diff. | oral cavity |
| HNX-P | well diff. | oral cavity |
| HNX-PV | (mucoepidermoid ca.) | oral cavity |
| HNX-SG | moderately diff. | hypopharynx |
| HNX-TI | well diff. | oral cavity |
| HNX-V | poorly diff. | larynx |
| HNX-W | moderately diff. | oral cavity |

*All tumours are squamous cell carcinomas except HNX-PV. Histology and site of origin in the patients from whom the xenografts were derived. For the sake of clarity, the initial characters HNX are omitted from the figures.

**Figure 1** Reaction of head and neck cancer xenografts to CDDP and BLEO. Growth delay is expressed in terms of the mean number of volume doubling times gained by the treatment. Difference between treated and control group from 100 to 200 and 400 mm³ ●: significant ($P<0.05$), ▲: not significant ($P>0.05$) and ■: completely regressed tumours in the treated group, no significance tested. CDDP: 3 mg kg$^{-1}$ daily for 3-5 days. BLEO: 15 mg kg$^{-1}$ daily for 4–7 days. The numbers of completely regressed tumours divided by the numbers of treated tumours are shown in the upper panels.

with all drugs: some lines were lost, while others are still in early passage. The maximum tolerated dose of CDDP caused a growth delay in all 6 lines treated (Figure 1.) The growth delay for tumour doubling varied from 1.4 in one line to a complete regression of all tumours in another line. BLEO, using a maximum tolerated dose, showed a significant growth delay in 6/7 lines (Figure 1). In 3/6 sensitive lines (HNX-G, -SG and -J) the regrowth of tumours was relatively late; for tumour growth from 100–400 mm³ a relatively long growth delay was seen.

MTX was tested using a schedule of 5 mg kg$^{-1}$ for 5–7 days till a maximum tolerated dose was reached. Only with one line (HNX-PV) was a small, but significant effect seen (Figure 2). With intention to mimic the situation in the clinic two high dose schedules were applied: (i) injection on Days 1 and 8 and (ii) injections on Days 1, 5 and 9. Toxicity studies with non tumour-bearing animals were

**Figure 2** Reaction of head and neck cancer xenografts to MTX. I. Daily injections of 5 mg kg$^{-1}$ for 5–7 days II. Injections on Days 1, 5 and 9. Mice with line W tumours received 50 and line B and line TI, 150 mg kg$^{-1}$ per injection. III. Injections on Days 1 and 8, 250 mg kg$^{-1}$ per injection, except lines KR and B which received 100 and 150 mg kg$^{-1}$ respectively.

performed to determine the $LD_{10}$ dose. It was found that the number of deaths did not correlate with the dose. Sometimes a high dose was less toxic than a lower dose. For instance, 100 mg kg$^{-1}$ MTX on Days 1 and 8 appeared to be more toxic than 250 mg kg$^{-1}$. Because of this variability it was decided to give relatively high doses (Figure 2). Another reason for the choice of these high doses was that the parallel toxicity study with a daily dose for 5 consecutive days showed that tumour-bearing animals appear to be less sensitive to MTX toxicity than non tumour-bearing animals (Table II). Using the schedule with injections on Day 1 and 8 no difference in toxicity could be found between tumour-and non tumour-bearing animals.

With these high dose schedules MTX did not cause a growth delay in any xenograft line (Figure 2). The effects of MTX on the tumours in two patients from whom xenografts were derived were known (Table III). Tumours of patients

**Table II** Toxicity of methotrexate in nude mice with and without tumour.

| | *Dose/injection (mg kg$^{-1}$)* | *Injections on Day* | *No. deaths/ total mice (%)* |
|---|---|---|---|
| Tumour bearing | 250 | 1,8 | 4/25[1] (16) |
| | 5 | 1–5 | 8/35[2] (23) |
| Non tumour-bearing | 250 | 1,8 | 1/7 (14) |
| | 5 | 1–5 | 5/7 (71) |

Groups of mice were injected i.p. The duration of the experiment was 40 days.
[1]Xenografts from 5 different lines.
[2]Xenografts from 6 different lines.

**Table III** Reactions to MTX of two head and neck tumours and their xenografts.

| *Xenograft line* | *Schedule* | *Growth delay xenograft (100–200 mm$^3$)* | *Treatment patient from whom xenograft was derived* | *Reaction patient* |
|---|---|---|---|---|
| HNX-TI | 5 mg kg$^{-1}$ Day 1–5 | 0.3 | 2 courses of MTX 24 h infusion i.a. followed by leucovorin-rescue. An interval of a week | 90% tumour regression |
| | 150 mg kg$^{-1}$ Days 1, 5 and 9 | 0.2 | | |
| | 250 mg kg$^{-1}$ Days 1 and 8 | 0.3 | | |
| HNX-W | 5 mg kg$^{-1}$ Day 1–5 | 0.3 | 4 courses of MTX 24 h infusion i.a. followed by leucovorin-rescue. An interval of a week | 50% tumour regression |
| | 50 mg kg$^{-1}$ Days 1, 5 and 9 | −0.2 | | |
| | 250 mg kg$^{-1}$ Days 1 and 8 | 0.3 | | |

Xenografts were obtained from the patients prior to chemotherapy.
i.a. = intra-arterial corresponding to lines HNX-TI and HNX-W regressed by 90% and 50% respectively after intra-arterial MTX treatment.

### Discussion

Xenografts of head and neck cancer can be grown succesfully in athymic nude mice and used for chemotherapy studies. This study shows that, using schedules with daily injections, CDDP was an effective agent; in all 6 xenograft lines a significant growth delay was found. BLEO was also effective; in only 1/7 lines was the growth delay insignificant. Clinical experiences with these agents have shown, that BLEO and CDDP produce a partial or complete remission in 38 and 26% of patients, respectively. (Taylor, 1979). The reason for the superiority of BLEO and CDDP against the xenografted tumours in comparison with the clinical data might be that in nude mice higher drug levels are attained. Another possibility is that only xenografts from sensitive patient tumours were tested since BLEO- and CDDP-insensitive head and neck cancer xenograft lines have been reported (Azar *et al.*, 1982). Although all but one of the tumour lines were sensitive to CDDP and BLEO a variation in growth delay was evident. For instance, CDDP caused complete regression of all tumours in one line and only a minimal growth delay in another. A better agreement with clinical findings could be obtained if those lines with a significant growth delay of less than two doubling times were considered insensitive in this model.

It is interesting to note that MTX gave only a minimal response. Tested against 11 lines, only in one was minimal growth delay observed. Also bolus injections, which simulated the clinical situation did not result in growth delay. Partial or complete remission have been reported for MTX in 40% (Carter, 1977) to 60% (Kirkwood *et al.*, 1981) of treated patients. It is therefore unlikely that only MTX-resistant tumours were implanted into the nude mice. Alternatively, more succesful xenograft take could have been characteristic of MTX-resistant tumours. The mechanisms responsible for this resistance are perhaps important for an increased tumorgenicity in nude mice.

It is interesting that for two tumours the reaction of the xenografts to MTX did not correlate with the source tumours which were sensitive in the patient. This experience is contrary to all reported comparisons, showing similar responses of the xenografts and the source tumours to the same treatment (Giovanella *et al.*, 1978, Nowak *et al.*, 1978, Fujita *et al.*, 1980, Shorthouse *et al.*, 1980). This observed lack of correlation in the response to MTX might be attributed to a difference in pharmacokinetics of MTX between man and mouse. At least the route of administration is different. The duration of exposure of xenografts to MTX may be too short. Experiments with more frequent drug administration will be carried out to test this possibility.

Resistance mechanisms potentially induced by xenografting may also account for the ineffectiveness of MTX in this model. The intracellular content of dihydrofolate reductase, the target enzyme to which MTX binds reversibly, may be increased or the rate of MTX uptake into the tumour cell may be decreased (Chabner, 1980). Another possibility is a reversal of MTX toxicity by exogenous purine and pyrimidine metabolites, released by a population of dying tumour cells. This cell loss could be higher in the xenografts than in the patient's tumour; a substantial part of the xenograft is often necrotic. This rescue mechanism can also be the basis of the protection against MTX toxicity by the presence of a tumour, as has been shown in the toxicity studies. With the low daily doses nucleic acid metabolites can play a role in protection against MTX toxicity since tumour (L1210)-bearing mice do not need purines to reverse MTX toxicity (Straw *et al.*, 1977). Further studies are needed to elucidate the cause of MTX ineffectiveness.

Toxicity studies of MTX reveal that the results are difficult to interpret. Toxicity appeared to be dependent on the presence of a tumour and, moreover, an $LD_{10}$ could not be determined because of large variability. The policy to give a daily dose for as long as the mice can tolerate it is an elegant solution in screening models to overcome the toxicity problem and is also economical in mice.

In conclusion, it is clear that response to CDDP and BLEO varies for the xenograft lines, as has been shown by other authors using other tumours and drugs (Bailey *et al.*, 1980, Houghton & Houghton, 1980 and Nowak *et al.* 1978). The lack of correlation between the xenograft and patient response to MTX appears to indicate that this model has certain limitations as a screening model for anticancer agents. It has yet to be established whether pharmacokinetic differences between man and mouse or resistance mechanisms in the xenograft itself are responsible for the lack of effect of MTX. It is also necessary that more direct comparisons between xenograft and patient response with the same agent are made.

This work was supported by the Queen Wilhelmina Cancer Foundation, grant no. AUKC-VU 82-11.
The authors thank the staff of the Laboratory of Experimental Medicine (Head, Dr. H.A. Brouwer) for the technical assistance, Dr. Ir. A.J. van Triet for the LDH-determinations, Dr. J. Lindenberger for the donation of two tumour lines, and Dr. A. Leyva for his critical reading of the manuscript.

### References

AZAR, H.A., FERNANDEZ, S.B., BROS, L.M. & SULLIVAN, J.L. (1982). Human tumor xenografts in athymic (nude) mice: chemotherapy trials in serially transplanted tumors. *Ann. Clin. Lab. Sci.*, **12**, 51.

BAILEY, M.J., GAZET, J.-C., SMITH, I.E. & STEEL, G.G. (1980). Chemotherapy of human breast-carcinoma xenografts. *Br. J. Cancer*, **42**, 530.

BRAAKHUIS, B.J.M., SNEEUWLOPER, G. & SNOW, G.B. (1983). The potential of the nude mice xenograft model for the study of head and neck cancer. *Arch. Otorhinolaryngol.* (In press).

CARTER, S.K. (1977). The chemotherapy of head and neck cancer. *Semin. Oncol.*, **4**, 413.

CHABNER, B.A. (1980). Antimetabolites. In: *Cancer Chemotherapy*, (Ed. Pinedo). Excerpta Medica: Amsterdam. p. 1.

DeCONTI, R.C. & SCHOENFELD, D. (1981). A randomized prospective comparison of intermittent methotrexate, methotrexate with leucovorin, and a methotrexate combination in head and neck cancer. Cancer, **48**, 1061.

FUJITA, M., HAYATA, S. & TAGUCHI, T. (1980). Relationship on chemotherapy on human cancer xenografts in nude mice to clinical response in donor patient. *J. Surg. Oncol.*, **15**, 211.

GIOVANELLA, B.C., STEHLIN, J.S., FOGH, J. & SHARKEY, F.E. (1978). Serial transplantation of human malignant tumors in nude mice and their use in experimental chemotherapy. In: *Proceedings of the Symposium on the use of Athymic (nude) Mice in Cancer Research*, (Eds. Houchens & Ovejera). New York: Fischer, p. 163.

HOUGHTON, J.A. & HOUGHTON, P.J. (1980). On the mechanism of cytotoxicity of fluorinated pyrimidines in four human colon adenocarcinoma xenografts maintained in immune-deprived mice. *Cancer*, **45**, 1159.

KIRKWOOD, J.M., CANELLOS, G.P., ERVIN, T.J., PITMAN, S.W., WEICHSELBAUM, R. & MILLER, D. (1981). Increased therapeutic index using moderate dose methotrexate and leucovorin twice weekly vs. weekly high dose methotrexate-leucovorin in patients with advanced squamous carcinoma of the head and neck. *Cancer*, **47**, 2414.

KOPPER, L. & STEEL, G.G. (1975). The therapeutic response of three human tumor lines maintained in immune-suppressed mice. *Cancer Res.*, **35**, 2704.

LINDENBERGER, J. (1981). Aspects of xenografted tumors of the ear, nose and throat. Morphology, cell kinetics, growth behaviour and immunology. In: *Thymusaplastic Nude Mice and Rats in Clinical Oncology*, (Eds. Bastert *et al.*). Fischer: Stuttgart. p. 449.

LOONEY, W.B., MAYO, A.A., ALLEN, P.M., MORROW, J.Y. & MORRIS, H.P. (1973). A mathematical evaluation of tumour growth curves in rapid, intermediate and slow growing rat hepatomata. *Br. J. Cancer*, **27**, 341.

NOWAK, K., PECKHAM, M.J. & STEEL, G.G. (1978). Variation in response of xenografts of colo-rectal carcinoma to chemotherapy. *Br. J. Cancer*, **37**, 576.

OSIEKA, R., HOUCHENS, D.P., GOLDIN, A. & JOHNSON, R.K. (1977). Chemotherapy of human colon cancer xenografts in athymic nude mice. *Cancer*, **40**, 2640.

PESCE, A.J., BUBEL, H.C., DIPERSIO, L. & MICHAEL, J.G. (1977). Human lactic dehydrogenase as a marker for human tumor cells grown in athymic mice. *Cancer Res.*, **37**, 1998.

POVLSEN, C.O. & JACOBSEN, G.K. (1975). Chemotherapy of a human malignant melanoma transplanted in the nude mouse. *Cancer Res.*, **35**, 2790.

SHORTHOUSE, A.J., SMYTH, J.F., STEEL, G.G., ELLISON, M., MILLS, J. & PECKHAM, M.J. (1980). The human tumour xenograft-a valid model in experimental chemotherapy? *Br. J. Surg.*, **67**, 715.

SHORTHOUSE, A.J., JONES, J.M., STEEL, G.G. & PECKHAM, M.J. (1982). Experimental combination and single-agent chemotherapy in human lung-tumour xenografts. *Br. J. Cancer*, **46**, 35.

SOKAL, R.R. & ROHLF, F.J. (1969). *Biometry*. p. 175. San Francisco: Freeman & Co.

STRAW, J.A., TALBOT, D.C., TAYLOR, G.A. & HARRAP, K.R. (1977). Some observations on the reversibility of methotrexate toxicity in normal proliferating tissues. *J. Natl Cancer Inst.*, **58**, 91.

TAYLOR, S.G. (1979). Head and neck cancer. In: *Cancer Chemotherapy; Annual* **1**, (Ed. Pinedo). Amsterdam: Excerpta Medica. p. 237.